# *MGMT* methylation analysis of glioblastoma on the Infinium methylation BeadChip identifies two distinct CpG regions associated with gene silencing and outcome, yielding a prediction model for comparisons across datasets, tumor grades, and CIMP-status

**DOI:** 10.1007/s00401-012-1016-2

**Published:** 2012-07-19

**Authors:** Pierre Bady, Davide Sciuscio, Annie-Claire Diserens, Jocelyne Bloch, Martin J. van den Bent, Christine Marosi, Pierre-Yves Dietrich, Michael Weller, Luigi Mariani, Frank L. Heppner, David R. Mcdonald, Denis Lacombe, Roger Stupp, Mauro Delorenzi, Monika E. Hegi

**Affiliations:** 1 Department of Clinical Neurosciences, Lausanne University Hospital, Lausanne, Switzerland; 2 Bioinformatics Core Facility, Swiss Institute for Bioinformatics, Lausanne, Switzerland; 3 Département de Formation et de recherche, Lausanne University Hospital, Lausanne, Switzerland; 4 Department of Neurology, Erasmus Medical Center, Rotterdam, The Netherlands; 5 Medical University of Vienna, Vienna, Austria; 6 University Hospital Geneva, Geneva, Switzerland; 7 Department of Neurology, University of Tübingen, Tübingen, Germany; 8 Department of Neurology, University Hospital Zurich, Zurich, Switzerland; 9 Department of Neurosurgery, Inselspital Berne, Berne, Switzerland; 10 Department of Neuropathology, University Hospital Zurich, Zurich, Switzerland; 11 Neurology and Neuro-Oncology, London Regional Cancer Program London Health Sciences Centre, University of Western Ontario, London, ON Canada; 12 EORTC Headquaters, Brussels, Belgium; 13 National Center of Competence in Research Molecular Oncology, ISREC-SV-EPFL, Lausanne, Switzerland; 14 Department of Neurosurgery, Laboratory of Brain Tumor Biology and Genetics, Centre Hospitalier Universitaire Vaudois (CHUV BH19-110), 46 rue du Bugnon, Lausanne, 1011 Switzerland

**Keywords:** MGMT, DNA methylation, MSP, Infinium methylation platform, Prediction model

## Abstract

The methylation status of the O^6^-methylguanine-DNA methyltransferase (*MGMT*) gene is an important predictive biomarker for benefit from alkylating agent therapy in glioblastoma. Recent studies in anaplastic glioma suggest a prognostic value for *MGMT* methylation. Investigation of pathogenetic and epigenetic features of this intriguingly distinct behavior requires accurate *MGMT* classification to assess high throughput molecular databases. Promoter methylation-mediated gene silencing is strongly dependent on the location of the methylated CpGs, complicating classification. Using the HumanMethylation450 (HM**-**450K) BeadChip interrogating 176 CpGs annotated for the *MGMT* gene, with 14 located in the promoter, two distinct regions in the CpG island of the promoter were identified with high importance for gene silencing and outcome prediction. A logistic regression model (MGMT-STP27) comprising probes cg1243587 and cg12981137 provided good classification properties and prognostic value (kappa = 0.85; log-rank *p* < 0.001) using a training-set of 63 glioblastomas from homogenously treated patients, for whom *MGMT* methylation was previously shown to be predictive for outcome based on classification by methylation-specific PCR. MGMT-STP27 was successfully validated in an independent cohort of chemo-radiotherapy-treated glioblastoma patients (*n* = 50; kappa = 0.88; outcome, log-rank *p* < 0.001). Lower prevalence of *MGMT* methylation among CpG island methylator phenotype (CIMP) positive tumors was found in glioblastomas from The Cancer Genome Atlas than in low grade and anaplastic glioma cohorts, while in CIMP-negative gliomas *MGMT* was classified as methylated in approximately 50 % regardless of tumor grade. The proposed MGMT-STP27 prediction model allows mining of datasets derived on the HM**-**450K or HM-27K BeadChip to explore effects of distinct epigenetic context of *MGMT* methylation suspected to modulate treatment resistance in different tumor types.

**Electronic supplementary material:**

The online version of this article (doi:10.1007/s00401-012-1016-2) contains supplementary material, which is available to authorized users.

## Introduction

High throughput platforms for genome wide DNA methylation analysis have allowed establishing the methylome of large series of patient samples. Pattern analysis of respective datasets has identified CpG island methylator phenotypes (CIMP) for several tumor types such as colon cancer [19, 21, 42] and more recently also glioma (G-CIMP) [29, 43, 45]. However, classification of samples as being silenced by aberrant methylation for a given gene is not obvious, since the relationship between CpG-methylation at individual sites, the extent of the overall CpG island methylation, and their effect on gene silencing is strongly dependent on the location within the gene [46]. Promoter methylation of the repair gene O^6^-methylguanine-DNA methyltransferase (*MGMT*) is a predictive factor for benefit from alkylating agent therapy in glioblastoma patients [3, 16, 33]. The predictive value of the *MGMT* status is supported by recent findings in two clinical trials, comparing radiotherapy versus temozolomide (TMZ) treatment. In these trials for elderly patients, retrospective analysis of the *MGMT* methylation status was associated with prediction of good outcome in the TMZ-, but not the RT-arm [24, 52]. Furthermore, the *MGMT* status has been prospectively validated in a phase III trial as biomarker for favorable outcome in glioblastoma patients treated with temozolomide [12]. Repair by MGMT reverses alkylation at the O^6^-position of guanine, one of the most toxic lesions induced by alkylating agents such as temozolomide (TMZ), thereby blunting the treatment effect [18, 30]. Hence, the *MGMT* methylation status has become a biomarker used for patient stratification or patient selection in clinical trials for glioblastoma patients [12, 39, 50]. Surprisingly, recent studies in anaplastic glioma (WHO grade III) suggest a prognostic value [44, 51]. In order to investigate pathogenetic and epigenetic features associated with this intriguingly distinct behavior of anaplastic glioma compared to glioblastoma, it is of high interest analyzing large datasets of glioma for which DNA methylome data have been reported, and classifying them by their *MGMT* gene promoter methylation status for integration into multi-dimensional molecular and clinical data analysis. Several glioma datasets comprising methylome data obtained on the Infinium HumanMethylation27 (HM**-**27K) or HM-450K BeadChip that interrogate at single-nucleotide resolution over 27,000 or 485,000 methylation sites per sample, respectively, have become publicly available [7, 29, 43, 45]. The most comprehensive glioblastoma dataset with over 200 samples is from The Cancer Genome Atlas [29, 40], widely used for hypothesis generation and validation in brain tumor research [17, 48, 53]; however, the *MGMT* methylation status has not yet been annotated.

The objective of this study was to propose a model determining the probability of *MGMT* promoter methylation allowing classification into methylated and unmethylated samples based on CpG methylation data obtained on the widely used HM-450K or HM-27K BeadChip. The model can be applied to other datasets for example to further investigation of the relationship of *MGMT* methylation with CIMP and other molecular and clinical parameters. The basic idea was to train the model using methylation-specific PCR (MSP)-based classification that we have shown to predict favorable outcome in a homogenously and prospectively treated glioblastoma patient population and for which we have obtained HM-450K data [15, 16]. Standard treatment included the alkylating agent temozomoide (TMZ) concomitant with and adjuvant to radiotherapy [36, 38]. At the same time, this study allowed investigation of the relationship between location-specific CpG methylation, *MGMT* gene expression and outcome, supporting the mechanistic hypothesis that methylation-dependent gene silencing results in loss of expression and subsequently benefit from alkylating agent therapy in glioblastoma.

## Material and methods

### Patient samples and external data sets

DNA methylation profiles were established for 63 glioblastoma tissues from 59 patients and five non-tumoral brain tissues (epilepsy surgery). All glioblastoma patients were treated within a phase II or a randomized phase III trial [36, 38] and provided written informed consent for molecular studies of their tumors. The protocols were approved by the ethics committees at each participating center and the respective competent authorities. For this patient cohort (M-GBM) that served as training set, detailed clinical information, treatment [37] and molecular data was available, including gene expression data [26], and the *MGMT* methylation status based on classic methylation-specific PCR (MSP) [15, 16].

Four external glioma DNA methylation datasets associated with clinical information were used: the first, prospectively collected glioblastoma samples of a cohort of 50 patients (E-GBM) treated with combined chemo-radiotherapy with TMZ (Stupp protocol) for which HM-27K and methylation-specific pyrosequencing-based (MS-PSeq) *MGMT* methylation information was available [7] (see supplementary Figure S1 for location of five interrogated CpGs). The second dataset with HM-27K information consisted of 241 glioblastoma samples (TCGA-GBM, survival information available for 239 samples) and was downloaded from The Cancer Genome Atlas (TCGA) website (http://tcga-data.nci.nih.gov/tcga/tcgaHome2.jsp) [29, 40]. The third comprised HM-27K data from 67 anaplastic glioma (WHO grade III) (VB-Glioma-III) [45] of a cohort of homogenously treated patients, including *MGMT* methylation classification based on methylation-specific multiplex ligation-dependent probe amplification (MS-MLPA) (see Figure S1 for location of the 3 interrogated CpGs, probes used are described [44]). The fourth dataset comprised 71 low grade and anaplastic glioma samples (29 WHO grade II and 42 grade III) profiled on the HM-450K (T-Glioma-II/III) [43].

### DNA methylation analysis

DNA was isolated from frozen tissues whereof 1.0-μg DNA was converted by bisulfite using the EZ DNA Methylation™ Kit (# D5001 Zymo Research Corporation) according to the manufacturer’s instructions. DNA methylation analysis was performed on the HM-450K (Illumina) as recommended at the Genomics platform at the University of Geneva. For each interrogated CpG two site-specific probes are present, one designed for the methylated and another for the unmethylated locus to which the chemically converted DNA gets hybridized. Single-base extension of the hybridized probes incorporates a labeled ddNTP, which allows subsequent quantification of methylated and unmethylated alleles (http://www.illumina.com/technology/infinium_methylation_assay.ilmn).

### Data analysis

The intensities of methylated and unmethylated signals were normalized using the Illumina GenomeStudio program. In the annotation file 176 CpG probes were associated with the *MGMT* gene, whereof 25 are shared with the HM-27K that was used in three of the four external datasets. The DNA methylation information was summarized by M-value as recommended by Du et al. [4]:

$$ {\text {M}\text{-}{\text {value}}= \log 2\left( {\frac{{\max \left( {{\text{signal B}},0} \right) + 1}}{{\max \left( {{\text{signal A}},0} \right) + 1}}} \right)}. $$

The terms ‘signal A’ and ‘signal B’ correspond to the intensities of the unmethylated and methylated probes.

### Statistical methods

**Fig. 1:**
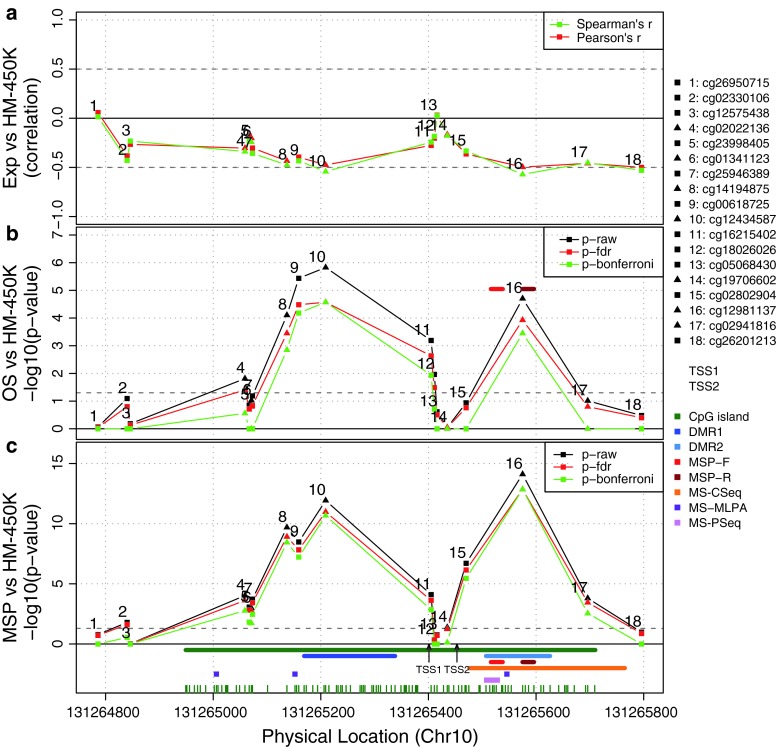
CpG methylation of the *MGMT* promoter region, *MGMT* expression and patient survival in M-GBM. **a** The Spearman and Pearson correlations between gene expression (probe 204880_at from Affymetrix U133plus2) and M-value of the 18 CpG methylation probes from the Infinium humanmethyltion 450K BeadChip (HM-450K) of the M-GBM cohort are visualized on a *scale* representing the physical location in the CpG island of the promoter region encompassing the transcription start site (TSS) (genome build 37). **b** The associations between overall survival (*OS*) and CpG methylation of distinct probes are displayed (*p* values, univariate Cox regression model and log-likelihood ratio test; *p* values, minus-log10-transformed). The *p* value for classification by MSP is indicated at its physical location (primer set, red). **c** The associations between *MGMT* promoter methylation classification based on MSP and the 18 selected CpG methylation probes from the HM-450K are shown (logistic regression and log-likelihood ratio test; *p* values, minus-log10-transformed). The *dotted gray* lines in **b** and **c** correspond to the threshold of 0.05. The *graph at the bottom* indicates the physical location of the TSS (TSS1, according to Harris et al. [14]; TSS2 according to gene build 19); the location of the CpG island/individual CpGs *green*; the differentially methylated regions 1 and 2, DMR1 and 2, as defined by Malley et al. [23] *blue*; the primers for MSP [6] *red*; the region for MS-clone sequencing in glioblastoma, MS-CSeq [34]; the CpGs interrogated by methylation specific multiplex ligation-dependent probe amplification, MS-MPLA *purple* [44], and methylation-specific pyrosequencing, MS-PSeq *pink* [7]. The names of the CpG probes interrogated on HM-450K are given on the right. Probes present on both platforms (HM-450K and HM-27K) are indicated by *triangles*, probes only present on the HM-450K are represented by *squares*. See supplementary Figure S1 for exact locations of CpGs interrogated by the different assays. The symbols are explained on the right hand side. We note that the CpG methylation probes (8, 9, 10 and 16) most correlated with expression also correspond to the probes highly associated with survival, and most correlated with MSP-based *MGMT* methylation prediction

The relationship between the methylation status of the *MGMT* promoter defined by MSP and the probes located in the *MGMT* promoter region (CHR10: 131264700-131266300, genome build 37) (Fig. 1; Fig. S1) present on the HK-450K was evaluated by logistic regressions [25, 28]. A two-step procedure was used to construct the model to calculate the probability of *MGMT* promoter methylation for subsequent binary classification. In the first step, all univariate models were tested and only probes significantly associated with the MSP classification were selected using the log-likelihood ratio test (LRT) and Bonferroni correction for multiple testing. In a second step, we determined the optimal model built by stepwise logistic model building based on the corrected Akaike’s criterion (AICc) [1, 20, 47] that limits overfitting. Two models were constructed, one with all selected probes, and one using only the probes common to both, the HM-27K and HM-450K platforms. The model performance was assessed by internal-validation based on a bootstrap procedure with optimism/bias correction [13]. This procedure validates the process used to fit the original model and it provides a bias value defined by the difference between the index from the original dataset and the average of indices from the resampling procedure (200 repetitions). The kappa index and the proportion of correct classification were used to evaluate the concordance between the observed and the predicted methylation status. The M-value distributions of the probes selected by the model were compared by pairwise quantile-quantile representation (QQ-plot) for the five datasets and non-parametric Smirnov–Kolmogorov test for equal distribution.

For the external datasets, the probability of *MGMT* promoter methylation and subsequent classification were determined using the model compatible with the HM-27K platform. The probability values were associated with Wald-based confidence intervals (CI 95 %) to evaluate the uncertainty of the model [9]. CIMP positive tumors were identified using unsupervised clustering methods similar to Noushmehr et al. and Turcan et al. [29, 43]. The relationship between the predicted *MGMT* status and the presence of CIMP was assessed by Chi-squared tests with *p* values computed by Monte Carlo simulation, because cell counts were expected to be inferior to five [31].

The Kaplan–Meier (KM) curves and log-rank tests were estimated for each dataset and predictor [13, 41]. Univariate and multivariate survival models were assessed using the Cox proportional hazards regression model and the LRT.

Analyses and graphical representations were performed using R-2.14.2 and the R packages design and survival [32].

## Results

### Calibration model to predict the methylation status of the *MGMT* promoter using the HM-450K platform

The prediction model is based on a glioblastoma patient cohort treated with standard chemo-radiotherapy within two prospective clinical trials. The *MGMT* promoter methylation status of this cohort was available from classic MSP discriminating methylated and unmethylated samples. Most importantly, the results of this MSP-test have been shown to be of predictive value for benefit from the addition of temozolomide chemotherapy [15, 16, 26, 37]. The results of the MSP assay were considered as reference for model construction. A total of 63 glioblastoma samples (M-GBM) and five non-tumoral brain tissues were analyzed on the HM-450K Beadarray platform. The five non-tumoral brain tissues were classified as *MGMT* unmethylated based on MSP. The clinical and molecular information of patient samples is summarized in Table S1. Of the 176 CpG probes on the HM-450K annotated as mapping to the *MGMT* gene, 14 are in the CpG island located in the *MGMT* promoter region encompassing the transcription start site (TSS) (Fig. 1). This CpG island has been shown to be determinant for regulation of expression [8, 23, 27, 35]. The remaining CpGs are mostly found in the *MGMT* gene body (Supplementary Figure S2). The detection call rate for these 14 CpG island-associated probes was equal to 1.00, indicating reliable detection.

### Association of probes with MSP, *MGMT* expression and outcome

Eleven probes located in the TSS-encompassing CpG island of the *MGMT* promoter were significantly associated with the previously MSP-defined *MGMT* methylation status (Fig. 1). We observed two prediction peaks. The strongest association was reached by the probes **cg12434587** and **cg12981137**, respectively (No. 10 and 16 in Fig. 1). Of note, the CpG tested by the probe **cg12981137** (CHR 10: 131265575, genome build 37) is also interrogated by the reverse primer of the MSP assay (Supplementary Fig. S1) [6]. Strikingly, the highest negative correlation between methylation and gene expression (estimated by Affymetrix U133plus2, [26]) was observed for the same two probes (Spearman correlation coefficients −0.543 and −0.571, respectively). The negative correlation is consistent with promoter methylation mediated down-regulation of *MGMT* expression. Similarly, these two probes were the most negatively correlated with *MGMT* gene expression in the E-GBM and the TCGA-GBM dataset for which expression data were available (Supplementary Fig. S3). The association of *MGMT* expression and DNA methylation at individual CpGs is visualized in a scatter plot for the M-GBM data set including non-tumoral brain tissues in the Supplementary Figure S4. Remarkably, the probes in vicinity to the TSS (probes 12–14 in Fig. 1) were at best weakly associated with the MSP results (Fig. 1). The strongest association with overall survival (OS) peaked at the same two probes, **cg12434587** and **cg12981137**, with *p* values of <10^−5^ and <10^−4^, while again no association was found at CpGs in the vicinity of the TSS, and the 5′ and 3′ edges of the CpG island (Fig. 1). The *p* values for OS prediction of these two probes were similar to the one for the MSP-based methylation classification (*p* < 10^−5^). The association of OS with methylation at individual CpGs is shown for all datasets in Figure S3. Taken together, the CpG methylation probes (8, 9, 10 and 16) most correlated with expression also correspond to the probes highly associated with survival, and most correlated with MSP-based *MGMT* methylation prediction.

In order to test if other methylated CpGs are relevant for *MGMT* silencing, we investigated the remaining CpGs, mainly located in the gene body. No negative correlation was observed between methylation and expression and no association with outcome (Supplementary Figure S2).

### Stepwise model building

We aimed at building an optimal methylation predictor using multiple probes. In the first step, the 11 probes significantly associated with MSP-defined methylation were selected. The prediction performances with individual probes were very high. Expectedly, the highest sensitivity and specificity values were obtained for the probes cg12981137 and cg12434587 (0.906 and 0.944; 0.875 and 0.944) (Fig. 1). Nonetheless, stepwise model building (see “Methods”) indicated that MSP assay classification could be predicted even better using two or more probes.

**Fig. 2:**
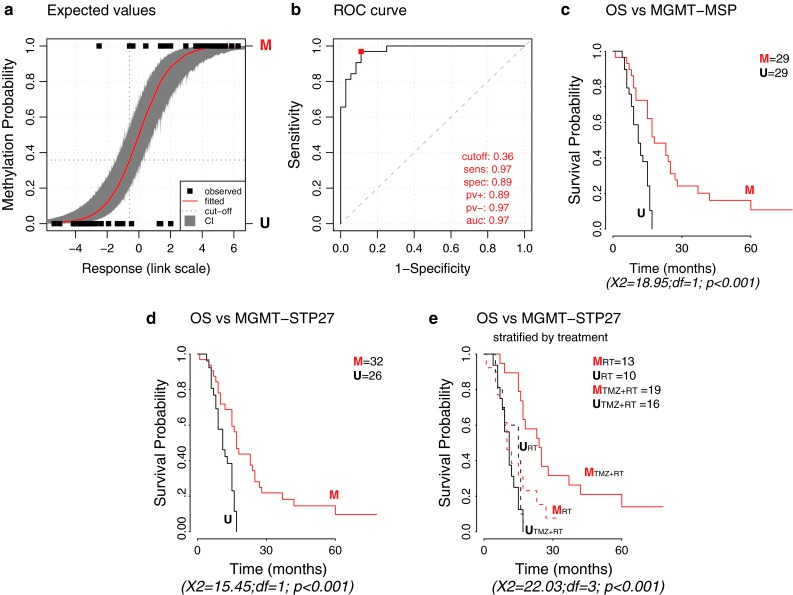
Performance of the stepwise logistic regression model (MGMT-STP27) for prediction of methylation status of the *MGM*T promoter. **a** Displays the estimated probability of methylation against the logit-transformed response fitted for the M-GBM dataset. The observed values are given by *full black squares*, indicating same or different classification by STP27 or MSP. Fitted values and their confidence intervals [CI] at 95 %, estimated by simulation, correspond to the *red line* and *gray area*, respectively. *Dark green dotted lines* indicate the threshold used to define methylated and unmethylated samples. **b** The receiver operating characteristic (*ROC*) curve is provided, where sensitivity (true positive rate) is plotted against 1-specificity (false positive rate). Accuracy is measured by the area under the ROC curve (AUC). Performance criteria are given for the optimal cut-off below the curve: optimal cut-off, sensitivity (*sens*), specificity (*spec*), positive predictive value (*pv+*), negative predictive value (*pv−*) and area under the curve (*auc*). The Kaplan–Meier curves for 58 patients are displayed for MSP-based classification (**c**) for the predicted methylation status obtained by the MGMT-STP27 model (**d**), and with additional stratification by treatment arm (RT + TMZ or RT treatment arm) (**e)** to visualize the predictive value of *MGMT* methylation for benefit from TMZ. Results of log-rank tests are given below each survival representation. *M* methylated, *U* unmethylated

The best model based on probes shared with the HM-27K platform (see Fig. 1) comprised the two probes cg12434587 and cg12981137 (MGMT-STP27 model). The adjustment of the model was of high quality (supplementary Table S2). The sensitivity and specificity were equal to 0.969 and 0.889, respectively (Fig. 2). The proportion of correct classification (0.926), the kappa index (0.853), and the AUC (0.974) confirm the good performance of the model. The hazard risks (HR) based on MSP and the MGMT-STP27 model were similar (log-rank test for both, *p* < 0.001; MSP, HR = 0.229, 95 % confidence interval (CI 95 %) = [0.115, 0.454]; MGMT-STP27, HR = 0.277, CI 95 % = [0.145, 0.529]) (Fig. 2). The equation for this model is given below:

$$ {{\text{logit}}\left( y \right) = 4.3215 + 0.5271 \cdot cg12434587 + 0.9265 \cdot cg12981137}. $$

The methylation probability *y* can be computed using the inverse logit function. For classification, we used a probability cut-off of 0.358, which empirically maximized the sum of sensitivity and specificity (supplementary Table S2; annotation of M-GBM sample classification supplementary Table S1). The classification by STP27 and MSP is visualized in Fig. 2a. Furthermore, the classification by STP27 is projected onto the scatter plots comparing expression and DNA methylation at individual CpGs, reclassification from MSP-based prediction are indicated (Supplemental Figure S4).

The second model, obtained using all 11 probes available on the HM-450K platform contained 4 probes: the two probes also selected in MGMT-STP27 (cg12981137, cg12434587) plus cg02022136 and cg23998405. The improvement over MGMT-STP27 is only marginal (inferior to 1 unit of the AICc, supplementary Table S2). However, the latter model showed a high inflated variance factor (8.9, supplementary Table S2) that reflects a problem of multi-colinearity. Consequently, this model was not considered for further analyses.

The internal validation based on the bootstrap procedure showed that the model MGMT-STP27 was relatively stable. The unbiased diagnostic accuracy (proportion of correct classification) was estimated to 91.24 %, against 92.65 % initially computed on the original dataset. For the kappa index, the difference between unbiased and original value was only equal to 0.03 units (supplementary Table S3).

### External validation of MGMT-STP27

**Fig. 3:**
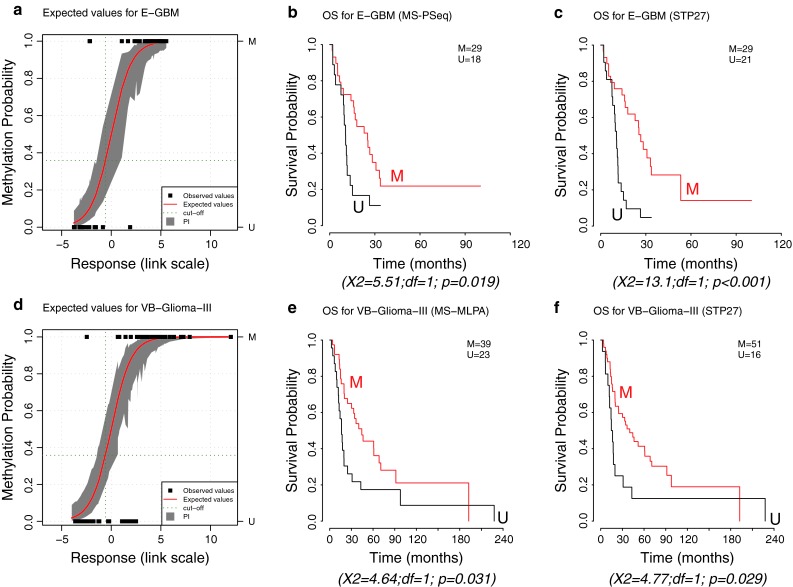
Validation of MGMT-STP27 in external datasets. The plots **a** and **d** represent the estimated probability of methylation against logit-transformed response fitted for the E-GBM, and VB-Glioma-III datasets using STP27. Fitted values and their prediction intervals [PI] at 95 %, estimated by simulation, correspond to the *red line* and *gray area*, respectively. *Dark green dotted lines* indicate the threshold used to define methylated and unmethylated samples according to STP27. The observed values are visualized by *full black squares*, indicating same or different classification by STP27 or MS-PSeq for E-GBM and MS-MLPA test for VB-Glioma-III, respectively. The Kaplan–Meier curves are based on classification by MS-PSeq for E-GBM (**b**), the MS-MLPA test for VB-Glioma-III (**e**), and based on the respective predicted methylation status using MGMT-STP27, in (**c**, **f**). Results of log-rank tests are given below each survival representation. *M* methylated, *U* unmethylated

We validated the use of the MGMT-STP27 model in an external data-set of 50 glioblastoma (E-GBM) analyzed on the HM-27K [7]. We dichotomized the MS-PSeq information of the *MGMT* promoter available for 47 cases into methylated and unmethylated. The cut-off was estimated at 7.28 % average methylation based on a fitted regression model visualized in supplementary Figure S5 which is similar to previous reports applying a cut-off of 8 % using the same MS-PSeq kit [10, 33]. The correspondence between the predicted status of *MGMT* promoter methylation using MGMT-STP27 and MS-PSeq information was very high, as visualized in Fig. 3a with a proportion of correct classification of 0.936, a kappa index of 0.875, and sensitivity and specificity equal to 0.931 and 0.944, respectively. Outcome prediction using MS-PSeq information or MGMT-STP27 was as follows: *p* = 0.019, HR = 0.454, CI 95 % [0.232; 0.891] for MS-PSeq, and *p* < 0.001, HR = 0.305, CI 95 % = [0.156; 0.596] for MGMT-STP27 (Fig. 3).

Next, we tested the model in a dataset from a cohort of anaplastic oligodendroglioma and anaplastic oligoastrocytoma (WHO grade III) (VB-Glioma-III, *n* = 67) generated with the 27K-platform [45]. The methylation status of the *MGMT* promoter was available from MS-MLPA for most cases (*n* = 62). We observed a good concordance (Fig. 3d) with a good classification rate of 0.839, a kappa index of 0.628, and sensitivity and specificity equal to 0.975 and 0.609, respectively. As reported by van den Bent et al. [45], *MGMT* methylation as determined by MS-MLPA was significantly associated with favorable outcome of the patients with a *p* value of 0.031 (log-rank test) (HR = 0.527, CI 95 % = [0.292, 0.952]). This is similar to the prediction by our MGMT-STP27 model with a *p* value of 0.029 (HR = 0.500, CI 95 % = [0.266, 0.941]) as visualized in Fig. 3.

### Prediction of *MGMT* methylation status in TCGA-GBM cohort and a glioma grade II and III data set

**Fig. 4:**
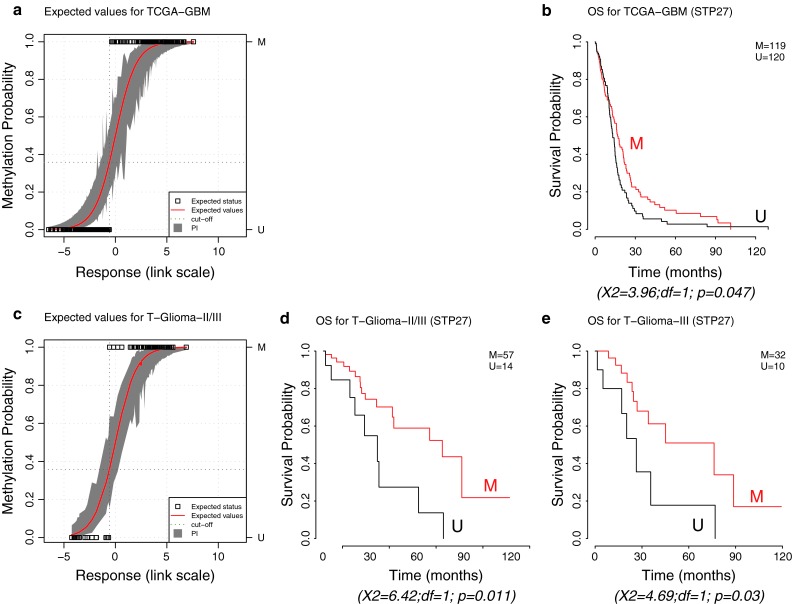
MGMT-STP27 based prediction in external datasets. The first plots in (**a**) and (**c**) represent the estimated values (probability of methylation fitted against response fitted in link space) for the GBM-TCGA and T-Glioma-II/III datasets. Fitted values and their prediction intervals [PI] at 95 %, estimated by simulation, correspond to the *red line* and *gray area*, respectively. *Dark green dotted lines* indicate the threshold used to define methylated and unmethylated samples. The *white squares* correspond to the deduced methylation status. The Kaplan–Meier curves are based on classification by prediction using MGMT-STP27 for TCGA-GBM (**b**), T-Glioma II/III (**d**), or only T-Glioma-III (**e**), Results of log-rank tests are given below each survival representation. *M* methylated, *U* unmethylated

The good performance of the MGMT-STP27 model in the two external data-sets suggests that we can appropriately predict the *MGMT* methylation status using common probes between the HM-450K and HM-27K platforms. Prediction of the *MGMT* methylation status in 241 glioblastoma available from TCGA (TCGA-GBM) with HM-27K data revealed a methylation frequency of 50 % (120/241) (see annotation of samples in supplementary Table S4 that also includes more recent samples analyzed on the HM-450K platform) similar to our M-GBM cohort. Patients from the TCGA-GBM dataset (*n* = 239) with a *MGMT* methylated glioblastoma had a more favorable outcome (*p* = 0.047, log-rank test, Fig. 4). The HR for the predicted methylation status was equal to 0.737 (CI 95 % = [0.545, 0.997]).

The prediction of the *MGMT* methylation status using MGMT-STP27 in a cohort of grade II and III gliomas (T-Glioma-II/III) with HM-450K data, determined a methylation frequency of 79 % (32/42) in grade III glioma and 86 % (25/29) in grade II. The favorable outcome associated with *MGMT* methylation was confirmed as visualized in Fig. 4.

### Associations of *MGMT* methylation across tumor grades of glioma

**Fig. 5:**
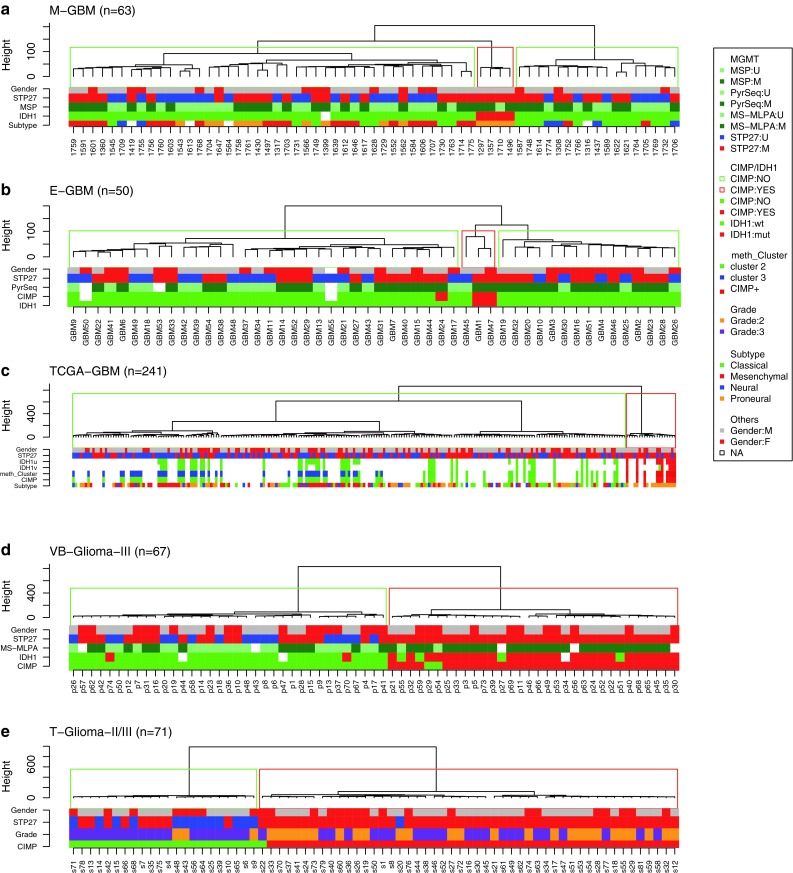
Distribution of *MGMT* methylation and CIMP status. The dendrogram for each dataset is provided. The five datasets were centered and normalized by probes followed by unsupervised hierarchical classifications of the 1,000 most variable probes (autosomes only) using Ward’s algorithm and Euclidean distance to establish CIMP classification (*green rectangle* for non-CIMP and *red* for CIMP). The methylation status of the *MGMT* promoter predicted by MGMT-STP27, *blue* for unmethylated, and *red* for methylated, is provided as label. Sample description comprise CIMP status as established in the respective original publication (if available), gender, IDH1 status (mutated or not, with additional annotation for TCGA-GBM; *u* unvalidated; *v* validated), classification into methylation clusters according to Noushmehr et al. [29] (cluster annotation Level 4 data, TCGA data portal), and gene expression based glioblastoma classification using a modified model from Verhaak et al. [48], tumor grade (for T-Glioma-II/III), and methylation status of *MGMT* promoter based on MSP, MS-MLPA, and MS-PSeq, unmethylated, *light green*; methylated, *darkgreen*. The color code for the labels is displayed

Next, we asked whether *MGMT* is part of the genes associated with CIMP using the TCGA-GBM data set [29]. Glioblastoma with a methylated *MGMT* promoter were significantly enriched among CIMP cases (15/20, 75 % against 105/221, 48 %; *p* = 0.023, Table S5). However, the two *MGMT* probes selected in our prediction model do not cluster with the CIMP core genes as visualized in the heatmap of supplementary Figure S6. Classification of the glioblastoma into the three methylation clusters (CIMP+, and 2 non-CIMP clusters) as published by Noushmehr et al. [29] were available for 81 samples with HM-27K DNA methylation (Fig. 5, Supplementary Fig. S6). The 10 CIMP+ samples defined by the authors were all in the CIMP cluster obtained by unsupervised clustering methods in the present study. The distribution of samples with methylated *MGMT* was not significantly different for the two non-CIMP methylation clusters (*p* = 0.214, Monte Carlo simulation) defined by Noushmehr et al. [29].

No association was found between the *MGMT* status and any of the expression-based glioblastoma subtypes proposed by Verhaak et al. [48] (*p* = 0.518, Monte Carlo simulation) (Fig. 5; Table S5).

**Fig. 6:**
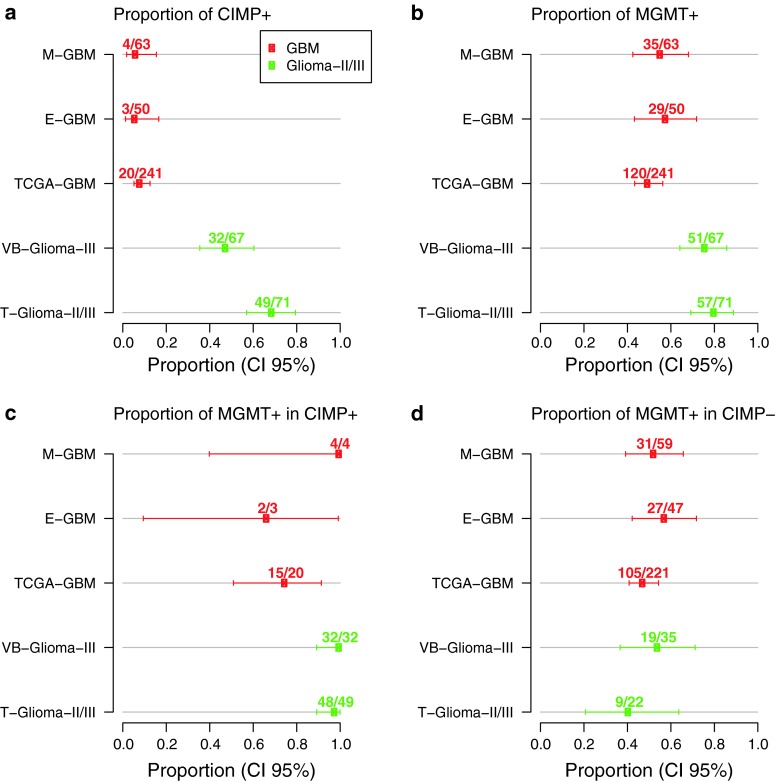
Proportion of predicted *MGMT* promoter methylation in CIMP+ or CIMP− gliomas. For all five glioma datasets, the proportion of CIMP+ (**a**), the proportion of *MGMT* methylation (**b**), and the proportion of *MGMT* methylation in CIMP+ (**c**) and CIMP− (**d**) tumors, respectively, are given. The CIMP status and the *MGMT* promoter methylation status are derived from unsupervised classification and MGMT-STP27, respectively

In contrast, in both the VB-Glioma-III and the T-Glioma-II/III datasets basically all tumors clustering together displaying CIMP were classified as *MGMT* methylated by the MGMT-STP27 model (32/32 and 48/49), while the CIMP-negative tumors exhibited a methylation frequency of 54 % (19/35) and 50 % (11/22) that is similar to the three glioblastoma datasets (Figs. 5, 6). Hence, the association of methylated *MGMT* with CIMP was much stronger in low grade and anaplastic glioma than in the TCGA-GBM. The difference is highly statistically significant (Chi-squared test with *p* value estimated by Monte Carlo simulation; 32/32 vs. 15/20, *p* < 0.005 for VB-Glioma-III and 48/49 vs. 15/20, *p* < 0.005 for T-Glioma-II/III). In the VB-Glioma-III dataset this association was retained when using the CIMP classification published by van den Bent et al. (*MGMT* methylated among CIMP+, 30/30; Fig. 5).

### Comparisons of M-value distribution across datasets and platforms

The pairwise comparisons of the M-value distribution for the two probes cg12434587 and cg12981137 across datasets revealed that M-value were generally higher in the grade II/III glioma (VB-Glioma-III, T-Glioma-II/III) as compared to glioblastoma (M-GBM and TCGA-GBM) (*p* < 0.05 from Kolmogorov–Smirnov tests, supplementary Figure S7). However, the comparisons between the glioblastoma datasets, analyzed on the HM-450K (M-GBM) and the HM-27K (TCGA-GBM), respectively, showed similar M-value distributions (*p* = 0.260 and *p* = 0.145, supplementary Figure S7). Likewise, the M-value distributions among the datasets of grade II and III gliomas analyzed on the HM-27K (VB-Glioma-III) and the HM-450K (T-Glioma-II/III), respectively, were similar (*p* = 0.435 and *p* = 0.233, supplementary Figure S7). Hence, indicating that differences observed between the studies were not due to a platform effect, but rather result from biological differences. Consequently, this could affect (bias) the prediction quality of the methylation status of *MGMT* for grade II and III glioma by MGMT-STP27, and may explain the higher number of positive calls by MGMT-STP27 when compared to MS-MLPA (specificity of only 0.609 when using MGMT-STP27 as predictor of MS-MLPA methylation calls). However, outcome prediction was equally good for both methylation call methods in the VB-Glioma-III data (Fig. 3).

## Discussion

The analysis of the *MGMT* gene in glioblastoma using HM-450K methylation data has shown a strong CpG location-dependent effect on patient outcome. To our knowledge, this is the first report describing the spatial relationship of CpG methylation in the *MGMT* promoter and the gene body and outcome of patients treated with alkylating agent therapy. Two regions of methylated CpGs with strong association to patient survival were identified (*p* < 0.0001 after Bonferroni’s correction) that are separated by a prediction minimum at the TSS. The two identified regions were also associated with the strongest negative correlation to *MGMT* gene expression, consistent with CpG methylation-mediated gene silencing and consequent sensitization to alkylating agent therapy due to lack of MGMT-mediated repair in this homogenously treated patient population. The two regions identified encompass the differentially methylated regions 1 and 2 (DMR1 and 2, Fig. 1) proposed by Malley et al. [23] to be most relevant for gene silencing when methylated in glioblastoma cell lines and xenografts. Shah et al. [35] defined three relevant regions, of which R2 and R3 encompass DMR1 and 2, and methylation of two of these three regions were associated with favorable progression free survival in their population of 44 glioblastoma patients treated with RT and concomitant and adjuvant TMZ. Most importantly, the region interrogated for diagnostic purposes using MSP [5, 16] overlaps with the CpGs associated with best outcome prediction identified here. In contrast, none of the 161 CpGs interrogated outside the CpG island, located mostly in the gene body, showed an association with outcome, or a negative correlation with *MGMT* expression (Supplementary Figure S2). However, we cannot exclude that other CpGs may be more relevant as the once described here, since the BeadChip array does not interrogate all CpGs of the CpG island encompassing the *MGMT* promoter (Supplementary Fig. S1).

The CpG methylation probes present on the HM-450K and HM-27K BeadChip identified to be most relevant for gene silencing and outcome allowed construction of a model for prediction of the *MGMT* methylation status. The use of logistic regression provides a simple model to calculate the methylation probability for a new sample based on two probes. Its ability to compute confidence and prediction intervals [2, 20] may be of particular interest for treatment decisions for patients whose tumors display methylation probabilities close to the cut-off (Figs. 2, 3, 4). This allows application of a “safety margin” as we do in EORTC26082 (NCT01019434) that selects unmethylated glioblastoma patients only (cut-off set at lower bound of 95 % CI using a quantitative MSP assay [49]), since it omits TMZ, thereby limiting the risk to withhold TMZ in patients who may potentially profit from it. The model’s good performance is reflected in similar or improved prediction of OS as compared to the MSP-based classification or MS-PSeq-based prediction in the E-GBM validation set, in accordance with high values for good classification, kappa value, and sensitivity and specificity measures (Figs. 2, 3).

Our model can be used for both the HM-450K and the HM-27K BeadChip. The platform effect was very weak. A higher amplitude of the methylation signal was detected in the low grade and anaplastic glioma samples that may simply reflect the fact that in non-glioblastoma usually both *MGMT* copies are present, while in glioblastoma only one is methylated and the other one is lost due to the characteristic high frequency of deletions of chromosome 10 that reached 90 % in the M-GBM samples [22]. Consequently, the presence of two methylated *MGMT* copies will lead to an increase of the ratio methylated to unmethylated alleles. For the model, however, this may generate a bias in the estimation of *MGMT* methylation probabilities based on the MGMT-STP27 model for non-glioblastoma tumors. The estimation could be improved by determining new optimal parameters in this population. Nevertheless, despite these limitations classification using the MGMT-STP27-based outcome prediction of the VB-Glioma-III dataset was similar to the one reported by the authors who used another method of *MGMT* testing (Fig. 3). Prediction of the MGMT status in the TCGA-GBM confirmed a favorable outcome for patients with *MGMT* methylation, although the effect was weaker than in our homogenously treated cohort (M-GBM) and the E-GBM cohort in which all patients were treated with combined chemo-radiotherapy comprising TMZ. This is not surprising, since most patients in the TCGA cohort had not (yet) been treated according to the current standard of care of combined chemo-radiotherapy (collection before 2005), and many different types of therapy were reported for the patients in the respective annotation file [29].

The annotation of the *MGMT* status in the TCGA-GBM dataset according to MGMT-STP27 allowed determining that *MGMT* is not a CIMP gene in glioblastoma although CIMP tumors were more likely to be *MGMT* methylated. Further, the prevalence of *MGMT* methylated glioblastoma is not different in the two non-CIMP methylation clusters defined by Noushmehr et al. [29], nor are they enriched in any of the expression-based glioblastoma subtypes, suggesting that *MGMT* methylation is not associated with a particular pathogenetic mechanism involved in the development of de novo glioblastoma.

This is in contrast to grade II and III glioma (VB-Glioma-III and T-GliomaII/III) in which *MGMT* is methylated in basically all CIMP tumors according to our classification model. It has been proposed that *MGMT* methylation may represent an epiphenomenon of CIMP in the context of grade III glioma [45]. This association of CIMP with *MGMT* methylation may provide the key to understand why *MGMT* methylation is associated with a prognostic and not a predictive value for benefit from alkylating agent containing chemotherapy in anaplastic glioma as suggested by two independent clinical trials [44, 51]. Most of the VB-Glioma-III samples analyzed here in fact originate from one of these two studies and were characterized for CIMP [45]. Anaplastic gliomas with CIMP accumulate other known favorable prognostic factors such as mutations of the isocitrate dehydroxygenase (*IDH*) genes, 1p/19 co-deletions, and also *MGMT* promoter methylation, in addition a plethora of other methylated genes whose contribution to response to therapy remains to be explored and exploited. It has become clear that these CIMP-positive tumors represent a pathogenetically different disease driven by epigenetic alterations mediated in most cases by IDH1/2 mutations [11, 43]. Interestingly, non-CIMP anaplastic gliomas showed a *MGMT* promoter methylation frequency similar to glioblastoma. It remains to be seen if in this CIMP-negative patient subpopulation the *MGMT* status is predictive for benefit from alkylating agent therapy like in glioblastoma or has a prognostic value. This question can be addressed in the ongoing CATNON trial (EORTC 26053-22054; NCT00626990) for anaplastic glioma comparing radiation therapy with or without temozolomide. The same question applies to low-grade glioma where radiation versus temozolomide treatment is tested (EORTC 22033-26033, NCT00182819) and the role of CIMP and *MGMT* methylation will need to be dissected. Since the HM-450K BeadChip allows the use of paraffin-embedded tumors, comprehensive DNA methylation analysis of samples collected within these clinical trials has become feasible.

The proposed MGMT-STP27 *MGMT* classification model will allow investigation of distinct epigenetic features associated with *MGMT* silencing in the context of CIMP-positive or CIMP-negative gliomas by multidimensional analysis of respective molecular and clinical data. Such alterations likely modulate response to therapy and may be exploited for improvement of personalized therapy.

## Electronic supplementary material

Below is the link to the electronic supplementary material.

Supplementary material 1 (PDF 50.3 kb)

Supplementary material 2 (XLS 58 kb)

Supplementary material 3 (XLS 28 kb)

Supplementary material 4 (XLS 58 kb)

Supplementary material 5 (EPS 1332 kb)

Supplementary material 6 (EPS 1259 kb)

Supplementary material 7 (EPS 1627 kb)

Supplementary material 8 (EPS 803 kb)

Supplementary material 9 (EPS 5400 kb)

Supplementary material 10 (EPS 1637 kb)
