# Supplementary material for: MGMT methylation analysis of glioblastoma on the Infinium methylation BeadChip identifies two distinct CpG regions associated with gene silencing and outcome, yielding a prediction model for comparisons across datasets, tumor grades, and CIMP-status
Source: Acta Neuropathol. 2012 Jul 19;124(4):547–60. doi: 10.1007/s00401-012-1016-2 (PMC3444709; doi:10.1007/s00401-012-1016-2)
Supplement: Supplementary file 1 — Supplementary material 1 (PDF 50.3 kb) [file 401_2012_1016_MOESM1_ESM.pdf]

Acta Neuropathologica

Supplementary Information Bady *et al.*

Tables S1-S5

Figures S1 to S7

References

MGMT methylation analysis of glioblastoma on the Infinium methylation BeadChip identifies two distinct CpG regions associated with gene silencing and outcome, yielding a prediction model for comparisons across datasets, tumor grades, and CIMP-status

Pierre Bady, Davide Sciuscio, Annie-Claire Diserens, Jocelyne Bloch, Martin J van den Bent, Christine Marosi, Pierre-Yves Dietrich, Michael Weller, Luigi Mariani, Frank L Heppner, David R Macdonald, Denis Lacombe, Roger Stupp, Mauro Delorenzi, Monika E Hegi

On behalf of the investigators of the European Organisation for Research and Treatment of Cancer and the National Cancer Institute of Canada Clinical Trials Group.

P. Bady, D. Sciuscio, A.-C. Diserens, J. Bloch, R. Stupp, M. E. Hegi (correspondance)
Department of Clinical Neurosciences, Lausanne University Hospital, Lausanne, Switzerland

P. Bady, M. Delorenzi
Bioinformatics Core Facility, Swiss Institute for Bioinformatics, Lausanne, Switzerland

P. Bady, M. Delorenzi
Département de Formation et de recherche, Lausanne University Hospital, Lausanne, Switzerland

M. JJ. van den Bent
Department of Neurology, Erasmus Medical Center, Rotterdam, The Netherlands

C. Marosi
Medical University of Vienna, Vienna, Austria

P.-Y. Dietrich
University Hospital Geneva, Geneva, Switzerland

M. Weller
Department of Neurology, University of Tübingen, Tübingen, Germany

M. Weller
Department of Neurology, University Hospital Zurich, Zurich, Switzerland

L. Mariani
Department of Neurosurgery, Inselspital Berne, Berne, Switzerland

F. L. Heppner
Department of Neuropathology, University Hospital Zurich, Zurich, Switzerland

D. R. Mcdonald
Neurology and Neuro-Oncology, London Regional Cancer Program London Health Sciences Centre, University of Western Ontario, London, ON, Canada

D. Lacombe
EORTC Headquaters, Brussels, Belgium

M. Delorenzi, M. E. Hegi
National Center of Competence in Research Molecular Oncology, ISREC-SV-EPFL, Lausanne, Switzerland

M. E. Hegi
Department of Neurosurgery, Laboratory of Brain Tumor Biology and Genetics, Centre Hospitalier Universitaire Vaudois (CHUV BH19-110), 46 rue du Bugnon, Lausanne, 1011 Switzerland

Table S1. Clinical and methylation characteristics of the 68 samples from M-GBM dataset.

[see excel file]

Description:

The table contains methylation probability (MGMT-STP27 response) and predicted methylation status (STP27 class; U, unmethylated/ M, methylated) of MGMT promoter given by the model MGMT-STP27. The observed M-values of the 18 probes related to the *MGMT* promoter region and MSP results are given for each sample. Survival information, time (Time) and status (alive=0, death =1, missing value is equal to NA), is provided for primary GBM (PrGBM=1).

Table S2. Performance and correlation characteristics of training set (M-GBM)

[see excel file]

Description:

Pearson (rpearson) and Spearman (rspearman) correlations between gene expression (probe *204880_at* from Affymetrix U133plus2) and M-values from the HM-450K BeadChip are given for each probe contained in *MGMT* promoter region. Logistic regression models are described by number of parameter (p) and sample size (n), percentage of covariate patterns superior to 4.0 (CovPat); Information criteria (AIC), Aikake's criterion, corrected AIC (AICc), Schwarz's Bayesian criterion (BIC). Performance criteria are given for the optimal cut-off (CutOff): sensitivity (Sens), specificity (Spec); Kappa index (Kappa); rate of good classification (Accuracy). The Averaged Variance Inflated factor (MVIF) is given when the models contained at least two parameters.

Table S3. Internal-validation by bootstrap with optimism (bias) correction for the logistic regression model (MGMT-STP27).

Statistic*	initial	training	test	optimism	corrected.value
RMSE	0.2646	0.2410	0.2741	-0.0332	0.2978
Accuracy	0.9265	0.9165	0.9025	0.0140	0.9124
Kappa	0.8532	0.8386	0.8082	0.0303	0.8229
R2 (a+bx)	0.7191	0.7407	0.7034	0.0373	0.6819
Origin (a)	0.0080	0.0053	0.0229	-0.0176	0.0256
Slope (b)	0.9830	0.9902	0.9548	0.0354	0.9475

*The 68 samples are drawn with replacement and this process is repeated 200 times to obtain an average bias.

RMSE (cost functions), root mean square deviation; Accuracy, rate of good classification; Kappa, Kappa index "R2", discrimination indices; "Origin" and "Slope" refer to the R2, slope and intercept calibration factors [3].

Table S4. Methylation characteristics of the 241 samples from TCGA-GBM and 74 TCGA GBM samples profiled on the Illumina HM-450K platform.

[see excel file]

Description:

The table contains methylation probability (STP27 response) and expected methylation status (STP27 class; U, unmethylated/ M, methylated) of MGMT promoter given by the model STP27. The CIMP classification is obtained by CTWC procedure and GBM expression subtype is based on a modified model from [7]. The both datasets (HM-27K and HM-450K) were identified by the variable called "dataset".

Table S5. Association tests in GBM-TCGA dataset.

	Subtype	CIMP	IDH1_v (validated)	IDH1_u (unvalidated)	STP27	cg12434587	cg12981137	gender
**GBM Subtype**	-	0.001	0.005	0.001	0.518	0.888	0.482	0.314
**CIMP**	33.593	-	0.001	0.001	0.023	0.262	0.022	0.467
**IDH1_v**	13.016	36.722	-	0.001	0.673	1.000	0.671	0.374
**IDH1_u**	21.098	58.000	36.722	-	0.296	0.439	0.152	0.747
**STP27**	2.191	5.543	0.577	1.933	-	0.001	0.001	0.438
**cg12434587**	0.707	1.507	0.145	0.932	127.309	-	0.001	0.115
**cg12981137**	2.488	6.306	0.772	2.670	217.834	111.009	-	0.683
**gender**	3.665	0.617	1.689	0.208	0.716	2.779	0.208	-

The lower triangle contains the observed chi-squared values and the p-values obtained by Monte Carlo simulation are given in the upper triangle of the table.

GBM subtype, expression based, proposed by Verhaak et al., models adapted [7];CIMP, CpG island methylator phenotype; IDH1_v, *IDH1* mutation data validated in TCGA, *IDH1*_u, unvalidated in TCGA; STP27, *MGMT* methylation status from STP27 model; cg12434587and cg12981137, CpG probes used in STP27.

Legends to Supplementary Figures S1 to S7

Figure S1. Representation of CpGs located in the CpG Island of the *MGMT* promoter region interrogated by different tests. The representation (chr10: 131’264’949-131’265’710; UCSC/hg19) indicates the physical location of the Transcription Start Site (first, according to Harris et al. [4] and second according to UCSC/hg19); the translation start codon (according to UCSC/hg19); the location of the individual CpGs, green; the primers for MSP [1], red; CpGs interrogated by methylation specific multiplex ligation-dependent probe amplification; MS-MPLA, purple [6] and methylation-specific pyrosequencing, MS-PSeq, pink [2]. The coding sequence of the gene is indicated in italic, and the CpGs interrogated by the Infinium HM-450K probes are numbered according to the scheme depicted in Figure 1.

Figure S2. CpG methylation of the *MGMT* gene, *MGMT* expression, and patient survival in the training set, M- GBM. (**a**) The Spearman and Pearson correlations between *MGMT* gene expression (probe 204880_at from Affymetrix U133plus2) and M-values of the 176 CpG methylation probes annotated with *MGMT* on the Infinium platform HM-450K (genome build 37) are visualized on a scale representing the physical location of the region encoding *MGMT*. (**b**) The associations between overall survival (OS) and CpG methylation of distinct probes are displayed (univariate Cox regression model and log-likelihood ratio test; p-values minus-log10-tranformed). (**c**) The associations between *MGMT* methylation classification based on MSP and the 176 CpG methylation probes from HM- 450K are shown (logistic regression and log-likelihood ratio test; p-values are provided after minus-log10-tranformation). (**d**) CpG methylation probes are classified by type: distance from transcription start site (TSS) in base pairs, TSS1500, TSS200; 5’-UTR; Gene Body; 3’-UTR; and group: Island, Shelf, and Shore according to the Infinium annotation file. The dotted grey lines in **b** & **c** correspond to the threshold of p=0.05.

Figure S3. CpG methylation of the *MGMT* gene, *MGMT* expression, and patient survival. (a-c) The Spearman and Pearson correlations between CpG methylation and gene expression are visualized on a scale representing the physical location of the region encoding *MGMT* for the three GBM datasets. (d-h) The associations between overall survival (OS) and CpG methylation of distinct probes are displayed

for all 5 datasets (p-values, univariate Cox regression model and log-likelihood ratio test; p-values, minus-log10-transformed). The dotted grey lines correspond to the threshold of 0.05. The probes are numbered as in Figure 1.

Figure S4. Relationship between CpG methylation and *MGMT* expression in M-GBM dataset. (**a**) For each probe a scatter plot of CpG methylation (M-values) and *MGMT* expression as estimated by the Affiymetrix probe 204408_at (log_2-transformed; U133plus2) is shown. The numbers 1 to 18 correspond to the numbering of the probes in Figure 1. The classification of the individual samples by the STP27 model and MSP is visualized with a color code as indicated on the lower right side, methylated, M/M, red, unmethylated, U/U, blue; reclassified by STP27 to methylated from unmethylated, M/U, orange; to unmethylated from methylated, U/M, green; non tumoral brain (NTB), grey. The Loess regression is shown as a solid black line. The box plots visualize the *MGMT* expression of glioblastoma classified as methylated (red) and unmethylated (blue) according to STP27 (**b**) or MSP (**c**). *MGMT* expression of non tumoral brain is shown in grey. The mean expression of *MGMT* in methylated samples is significantly lower than unmethylated (p=0.002 for STP27-MGMT and p=0.003 for MSP-MGMT, p-values from t-tests). The mean expression of NTB (n=4) is 6.510 (CI95% = [6.272; 6.745]), which is different from methylated (6.22, CI95% = [6.109; 6.344]), but not different from unmethylated glioblastoma tissue (6.52, CI95% = [6.379; 6.657]). The method of classification does not influence the mean expression of methylated or unmethylated group of glioblastoma.

Figure S5. Determination of cut-off for *MGMT* testing by MS-Pyrosequencing in E-GBM dataset. The MS-PSeq information available for the E-GBM data set was processed as described [2]. The percentage of *MGMT* methylation was averaged over the 5 CpG-sites interrogated (PyroMark Q96 CpG MGMT kit Qiagen) (see location of CpGs in Fig. 1, Fig. S1). The data was dichotomized into unmethylated and methylated status using an iterative procedure based on segmented regression [5]. The optimal cut-off obtained was 7.28%, defined as the point where the sum of squares of residuals is minimal.

Figure S6. Heatmap representations of TCGA-GBM and CIMP. The values of the 1000 most variable CpG probes (autosomes only) of the TCGA-GBM dataset derived on the HM-27K served as input into the analysis. The data was centered and scaled by probes. The dendrograms were obtained by Ward's classification and Euclidean distance. The methylation status of the *MGMT* promoter obtained by the MGMT-STP27 model is annotated for each sample (Table S1). The sample description is complemented by information on CpG island methylator phenotype status (CIMP) as given by the clustering, or as annotated in the TCGA database, gender, *IDH1* status (mutated or not according to the TCGA annotation file; u, unvalidated; v, validated) and gene expression based GBM subtype, modified model from Verhaak et al. [7] The location of the two *MGMT* probes (cg12434587 and cg12981137) used in the MGMT-STP27 model is indicated on the right. The color code for the labels and the heat map are displayed.

Figure S7. Comparison of M-value distributions between datasets The M-values of the probes cg12434587 and cg12434587 used in MGMT-STP27 were compared by quantile-quantile representation (QQ-plot). The red line corresponds to the line y=x. The terms 'D' and 'p' refer to the comparison of distribution by the Kolmogorov-Smirnov test. The platform Illumina used is indicated for each dataset. When the p-value is inferior to 0.05, the two distributions are considered as significantly different. We observed that distributions between GBM datasets (M-GBM and TCGA-GBM) or the grade II and III glioma datasets (VB-Glioma-III; T-Glioma-II/III) were relatively similar. However, the observed values appear to be generally higher in the glioma II and III datasets than in GBM cohorts (M-GBM, TCGA). The platform does not seem to contribute to the observed difference.

References

1 Esteller M, Hamilton SR, Burger PC et al (1999) Inactivation of the DNA repair gene O6-methylguanine-DNA methyltransferase by promoter hypermethylation is a common event in primary human neoplasia. Cancer Res 59:793-797

2 Etcheverry A, Aubry M, de Tayrac M et al (2010) DNA methylation in glioblastoma: impact on gene expression and clinical outcome. BMC Genomics 11:701

3 Harrell FE (2001) Regression modeling strategies, with applications to linear models, survival analysis and logistic regression. Springer, New York

4 Harris LC, Potter PM, Tano K et al (1991) Characterization of the promoter region of the human O6-methylguanine-DNA methyltransferase gene. Nucleic Acids Res 19:6163-6167

5 Muggeo VM (2003) Estimating regression models with unknown break-points. Stat Med 22:3055-3071

6 van den Bent MJ, Dubbink HJ, Sanson M et al (2009) MGMT promoter methylation is prognostic but not predictive for outcome to adjuvant PCV chemotherapy in anaplastic oligodendroglial tumors: A report from EORTC Brain Tumor Group Study 26951. J Clin Oncol 9:5881-5886

7 Verhaak RG, Hoadley KA, Purdom E et al (2010) Integrated genomic analysis identifies clinically relevant subtypes of glioblastoma characterized by abnormalities in PDGFRA, IDH1, EGFR, and NF1. Cancer Cell 17:98-110
